# Fluorescent Bulk Waveguide Sensor in Porous Glass: Concept, Fabrication, and Testing

**DOI:** 10.3390/nano10112169

**Published:** 2020-10-30

**Authors:** Zhong Lijing, Roman A. Zakoldaev, Maksim M. Sergeev, Vadim P. Veiko

**Affiliations:** 1 Faculty of Laser Photonics and Optoelectronics, ITMO University, 197101 Saint Petersburg, Russia; zlj.itmo@gmail.com (Z.L.); mmsergeev@itmo.ru (M.M.S.); vpveiko@itmo.ru (V.P.V.); 2 School of Optical and Electronic Information, Huazhong University of Science & Technology, Luoyu Road 1037, Wuhan 430074, China

**Keywords:** porous glass, sensor, waveguide, laser direct writing, rhodamine 6G, small molecules, ethanol, fluorescence

## Abstract

In this work, we suggest the new concept of sensing elements—bulk waveguides (BWGs) fabricated by the laser direct writing technique inside porous glass (PG). BWGs in nanoporous materials are promising to be applied in the photonics and sensors industries. Such light-guiding components interrogate the internal conditions of nanoporous materials and are able to detect chemical or physical reactions occurring inside nanopores especially with small molecules, which represent a separate class for sensing technologies. After the writing step, PG plates are impregnated with the indicator—rhodamine 6G—which penetrates through the nanoporous framework to the BWG cladding. The experimental investigation proved the concept by measuring the spectral characteristics of an output signal. We have demonstrated that the BWG is sensitive to ethanol molecules captured by the nanoporous framework. The sensitivity of the peak shift in the fluorescence spectrum to the refractive index of the solution is quantified as 6250 ± 150 nm/RIU.

## 1. Introduction

Sensors are in great demand in the era of expanding human influence on Earth and in space [1]. Such progress comes along with a variety of technogenic threats, which should be prevented with proper detection [2,3]. Especially, the detection of trace amounts of small molecules which have a low molecular weight (<900 Daltons and with the size ~1 nm [4])—ethanol [5], acetone [6], formaldehyde [7], etc.—has become a major challenge for sensing technologies. Thus, novel principles and mechanisms for small molecules detection are highly demanded [8].

Recent years have demonstrated remarkable progress in the design and fabrication of optical porous glass (PG) sensors applied for monitoring and controlling different media and object parameters [7,9]. Basically, such sensors consist of three main parts: a light source, receiver, and primary transducer. The primary transducer is a PG plate, which stores indicators [7,10,11]; it is ready to absorb the target molecules. When the PG sensor is placed in an environment with target molecules, the primary transducer converts the chemical reaction occurring in nanopores into a measurable optical signal, for example, the absorption of radiation from the light source at a certain spectral range.

Y.Y. Maruo’s group has developed a series of gas sensors with PG storing organic indicators such as indigo carmine [12] and Schiff’s reagent [13]. Such sensors can effectively detect formaldehyde or changes of indoor ozone concentration as large analytical instruments do. In particular, the action of an environmental gas changes the PG color signalizing the excess of the target molecules in the atmosphere. Recently, our team provided direct laser-induced fabrication of molecular barriers in PG to integrate several indicators in a single plate [14]. However, the mentioned approaches using bulk PG plates impregnated with an indicator have several inherent shortcomings that hinder their further improvement in both efficiency and sensitivity. They are the following: (1) high insertion loss due to residual light scattering and reabsorption of light by indicator molecules; (2) long response time with a typical measurement interval of 30 min; and (3) complexity of combining the PG sensor with an optical fiber system.

To overcome the shortcomings, we propose a new configuration of a PG sensor based on the inscription of a three-dimensional micro-sized optical channel, namely bulk waveguide (BWG). Unlike the previous configuration of PG-based sensors, the indicator is introduced to the BWG cladding. The radiation transmitted through the BWG interacts with the indicator generating a fluorescence signal at the output. The fluorescence spectrum is sensitive to chemical reactions occurring in the nanoporous framework in the cladding. For example, organic molecules such as rhodamine 6G react with ethanol molecules, whose presence red-shifts the fluorescence peak [15]. This is the precise interrogation of chemical reactions occurring in the nanoporous framework that opens new ways for small molecules detection captured from fluids or gas phases.

Thus, the main advantages of this configuration are the following: (i) the BWG may have a higher response rate than a PG-based sensor. The reason is that the light–matter interaction length of the PG-based sensor is limited by the plate thickness of ~0.5 mm, while the light–matter interaction length of the BWG equals the length of the waveguide of ~10 mm or even higher. (ii) The BWG may have lower insertion losses than the PG-based sensor due to the quartz core of the BWG, which reduces optical losses. (iii) The BWG is compatible with fiber optic systems and is suitable for integration purposes.

Laser direct writing (LDW) has become an advanced photonic technology that currently represents an important tool for micro/nanoscale structuring in glass materials [16,17] including BWGs inscription. To date, only pre-surface waveguides have been fabricated in solid glasses [18,19] to estimate fluid properties, e.g., refractive index, deposited on the glass surface. LDW in PG was also utilized for various needs: multilevel data writing [20], plasmonic properties tuning [21], and micro-cavities formation [22]. Previously, our team proposed and investigated the femtosecond laser-induced PG densification mechanism [23], which can be easily adopted for BWGs inscription.

The aim of this work is to develop and demonstrate a BWG sensor in PG for ethanol molecules detection. We implemented three steps. The first is the fabrication of BWGs in PG, and investigation of their geometrical, morphological, and optical properties. In parallel, we prove the concept of spatial integration of BWGs in a single glass plate—a BWGs array consisting of 40 channels is fabricated. Second, BWG cladding is impregnated with rhodamine 6G molecules to provide the sensitivity to target molecules—ethanol liquid. Third, ethanol molecules captured by the nanoporous framework influence the spectral properties of the optical signal transmitted through the BWG.

## 2. Laser Procedure, Materials, and Test

### 2.1. Waveguides Fabrication

PG plates with the content of 0.30 Na_2_O, 3.14 B_2_O_3_, 96.45 SiO_2_, and 0.11 Al_2_O_3_ (wt.%), an average pore diameter of 4 nm, and porosity of 26% were applied for BWG inscription. For this, a typical direct laser writing station based on a Yb-doped fiber laser (ANTAUS-20W-20u/1M, Avesta, Moscow, Russia) with a linear polarized Gaussian laser beam operating at 1035 nm wavelength with a pulse duration of 220 fs and fixed repetition rate of 1 MHz was utilized. The objective (20X, 0.4, LOMO, St. Petersburg, Russia) provided the laser beam waist with a diameter (2w_0_) equal to 2.5 μm. The XYZ translation stage based on a stepper motor with the step equal to 1 µm and controlled by the driver (Avesta, SMC-AD3, Moscow, Russia) was applied to translate the PG sample. The range of regimes resulting in PG framework densification has been established by us previously [14,23,24], and here we apply the fastest one to fabricate BWGs. The BWG inscription occurred from the sample translating perpendicular to the focused laser beam axis at speed 3.75 mm/s and incident pulse energy E_p_ = 0.6 µJ. For the next step, both ends of the waveguide were polished for light coupling.

### 2.2. PG Impregnation Procedure

Next, a fluorescent solution fills the nanoporous framework of the PG with the waveguides inscribed. The fluorescent solution was prepared by dissolving rhodamine 6G powder (5 mg) (Lenreactiv, St. Petersburg, Russia) in 1 mL ethanol. Thus, the molar mass of rhodamine 6G molecules is 379.02 g/mol, and the concentration of this solution is ~1.044 × 10^−2^ mol/L. The rhodamine solution mixed with ethanol liquid in a specific ratio, and the concentrations of ~1.670 × 10^−3^ mol/L and ~0.835 × 10^−3^ mol/L were selected for comparisons. The impregnation was conducted by immersing PG in the prepared rhodamine solution, as the solution penetrates nanopores due to the capillary effect. Then, the additional heat treatment (100 °C, 0.5 h) in the furnace released ethanol molecules from the nanopores leaving the fluorescent molecules. The porous release is important for the following sensing step. Sample transmittance and reflectance were measured in the range from 0.4 to 0.8 μm by a spectrophotometer (MSFU-K Yu-30.54.072, LOMO, St. Petersburg, Russia), where the minimum photometrical region is equal to 2 µm.

**Figure 1:**
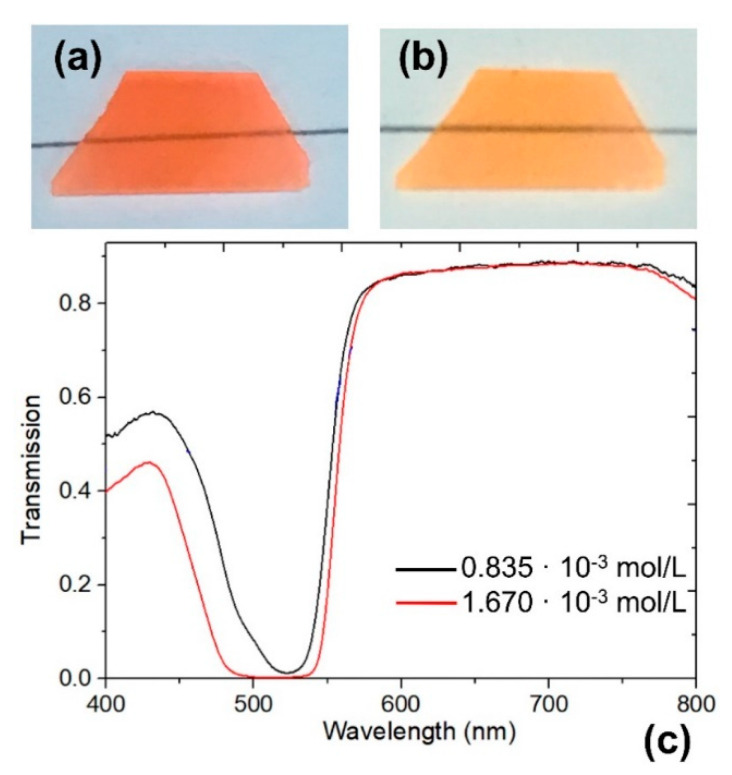
Photos of porous glass (PG) samples impregnated with rhodamine concentrations of (**a**) ~1.670 × 10^−3^ mol/L, and (**b**) ~0.835 × 10^−3^ mol/L. (**c**) Corresponding transmission spectrum in the visible spectral region of 400–800 nm.

Before the next step, we had to choose an appropriate concentration of rhodamine 6G in PG. Figure 1 shows photos and spectra of the prepared two samples with concentrations of the solution of ~1.670 × 10^−3^ mol/L (Figure 1a) and ~0.835 × 10^−3^ mol/L (Figure 1b). The absorption peak at ~523 nm wavelength of the sample with rhodamine concentration ~0.835 × 10^−3^ mol/L is a classic case of an impregnated porous silica material with a moderate rhodamine concentration [25]. The increase in rhodamine concentration up to ~1.670 × 10^−3^ mol/L results in a strong absorption in the range of 490–540 nm, which in the case of sensing will only reduce the sensitivity. Therefore, the lower concentration of the solution was chosen in this study. The inserted photo (Figure 1b) shows the top view of the PG impregnated with rhodamine 6G after the heat treatment. At such a distance, the sample shows the uniformity of impregnation.

### 2.3. Principle Operation of BWG Sensor and Testing

**Figure 2:**
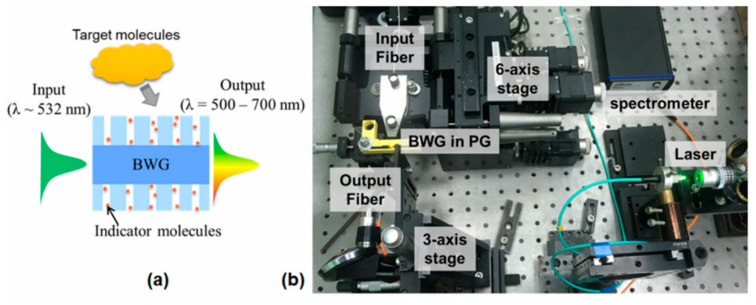
Scheme of the bulk waveguide (BWG) transducer concept (**a**). Experimental setup for testing the BWG transducer and the inserted photo of laser radiation coupling into the BWG by fiber-sample-fiber connection (**b**).

The proposed BWG transducer aims to detect the presence of ethanol molecules in the nanoporous framework of the PG. Figure 2a shows the schematic view of the BWG transducer following the concept:

(i) Platform: nanoporous optically transparent glass—PG—which captures and stores molecules of organic indicators, such as rhodamine 6G, thymol, and bromcresol;

(ii) Light guiding part: laser-written BWG, which possesses a core–cladding structure; it is located at the required depth of the PG plate. The cladding is sensitive to the environment actions, while the core has optical properties close to fused silica;

(iii) Spectral sensitivity: the nanoporous framework delivers indicator molecules to the BWG cladding. Here, we need an additional heat treatment that evaporates the fluid base (water or ethanol) of the indicator and frees the pore space for further capturing of target molecules from the glass surface;

(iv) Operation: the BWG confines the laser light by total reflection at the interface between the high and low refractive index of the core and cladding, respectively. Indicator molecules surround the core; we observe the fluorescence peaks with maximum signal intensity at the BWG output. For example, 532 nm laser radiation excites the indicator molecules. The excited molecules generate fluorescence in the range of 500–700 nm [15], which can be registered at the waveguide output by a fiber spectrometer. The concentration of the captured ethanol molecules affects the shift and intensity of the fluorescence peak.

According to the concept, we assembled the experimental setup for testing the BWG transducer, as seen in Figure 2b. The setup contains: (1) a fiber output laser with a center wavelength of 531.7 nm and FWHM bandwidth of ~0.4 nm (green diode laser); (2) input and output fibers with the BWG transducer in PG impregnated with rhodamine 6G placed between them; (3) an out-coupling fiber connected with a spectrometer (measuring range 220–770 nm and resolution 0.26 nm, AvaSpec-3648, Avantes, Apeldoorn, Netherlands); and (4) multiple-axis translation stages supporting precise control of fibers’ position.

## 3. Results and Discussion

### 3.1. Waveguide Properties

**Figure 3:**
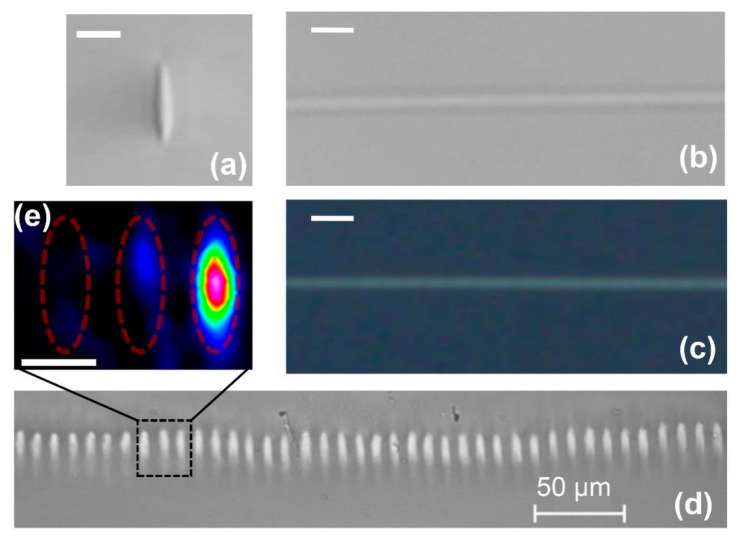
Microscopy investigation of BWGs: cross-section image (**a**) and corresponding top-view (**b**); the same top-view in crossed-polarized light (**c**); cross-section image of the BWGs array in a single PG plate (**d**); investigation of cross-talk between BWGs by the caption of intensity distribution at the BWG output (**e**). The scale bar is 10 μm.

Figure 3 presents the optical microscopy images of the BWG written by a femtosecond laser with pulse energy E_p_ = 0.6 µJ and a translation speed of 3.75 mm/s. All the BWGs are located at 300 µm depth below the PG surface. The fabricated BWGs are characterized by elliptical geometry, which is common for laser-written waveguides [26] and can be improved to produce almost symmetrically shaped waveguides according to [27]. The height equals ~12 µm and the width is about 5 µm (Figure 3a). The top-view image of the BWG in Figure 3b indicates a higher glass density across the waveguide. The investigation with polarized light, which uses a cross-polarizer pair, shows another top-view image in Figure 3c, where a bright central part of the BWG is visible. The bright light in the modified regions in PG is usually associated with the birefringence phenomenon [22] that confirms the anisotropic structure. In our case, observing the absence of bright light around the BWG cladding indicates the absence of lateral residual stresses around the BWG. This result is important from the point of view of prospects for spatial integration in PG, opening an opportunity to fabricate arrayed BWGs with low light cross-talk between neighbor waveguides in a single glass plate. We decided to prove that and fabricated a 40-channel BWGs array with a spacing of 10 μm (Figure 3d). The glass sample remained undamaged after recording such an array. In addition, at the stage of laser radiation coupling into the BWG, we observed a small amount (<5%) of radiation coupled in the neighbor BWG (Figure 3e), which demonstrates a low cross-talk in the BWGs array. It should be pointed out that the cross-talk is typical for waveguides in solid glasses [28], and it obligates the minimal spacing in the array to be equal to 40 μm [29].

**Figure 4:**
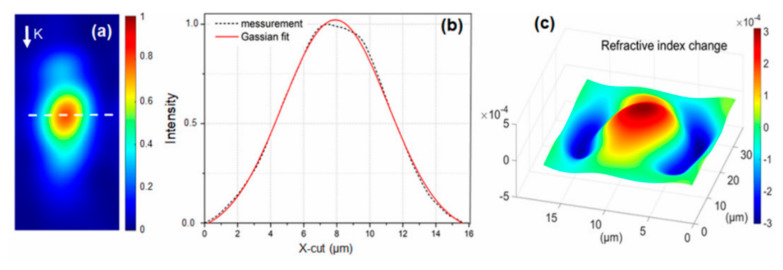
BWGs optical investigation results: near-field intensity distribution captured by CMOS camera (Gentec Beamage-3.0, QC, Canada) applying the objective (40×, 0.65 NA) at the BWG output (**a**). K represents the wave vector direction. The dashed line represents the X-cut position; *X*-axis intensity distribution of the BWG mode (**b**); the simulated refractive index contrast of the BWG cross-section (**c**).

The output intensity distribution of He-Ne laser radiation coupled in BWGs (Figure 4a) gives more details about the waveguide structure, which we were unable to resolve by optical microscopy. The distribution replicates the waveguide shape—an elongated ellipse. The full-width-at-half-maximum (FWHM) of the mode field diameter equals 7.0 × 11.0 μm. The Gaussian function fits well with the captured *X*-axis distribution with divination of ~6% (Figure 4b). The insertion losses of the waveguide are measured using a single-mode fiber butt-coupling method [30]. The averaged insertion losses are ~1.2 dB/cm at a wavelength of 975 nm.

Based on the mode profile, a numerical method, known as the refracted near-field method [31], is used to estimate the refractive index profile (Δn) of the BWG (Figure 4c). The BWG shows a “core–cladding” structure, where the cladding (negative Δn) wraps from both sides of the core (positive Δn). The maximum refractive index contrast between the core and cladding is ~6.0 × 10^−4^. The result obtained supports the concept (proposed in Section 2.3) of the BWG transducer: the core–cladding structure guides the light, and the cladding may capture the indicator molecules.

### 3.2. Fluorescence BWG Transducer: Proof of Concept

**Figure 5:**
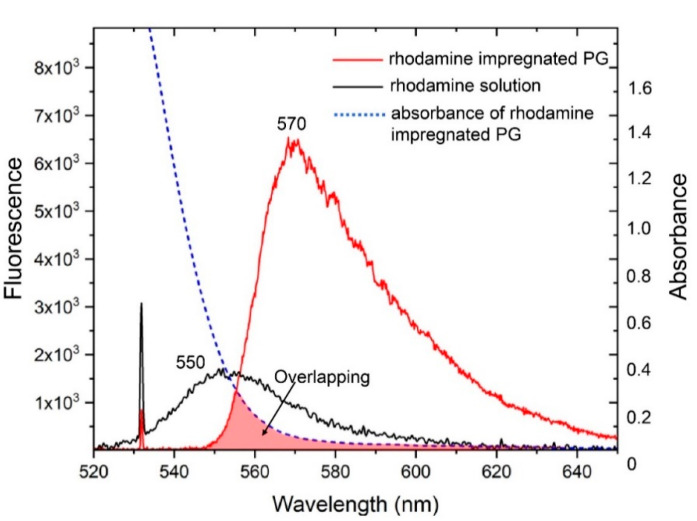
The spectrum curves of laser radiation transmitted through rhodamine 6G solution (0.835 × 10^−3^ mol/L) (black curve) and PG impregnated with the same solution (red curve). The absorbance spectrum of the rhodamine-impregnated PG, which was measured in Figure 1 (dashed blue curve) and applied here for the comparison.

We performed the spectral registration of laser radiation transmitted through the BWG in PG impregnated with rhodamine 6G on the setup (Figure 2b) to prove the concept of the BWG transducer detecting ethanol molecules. Figure 5 represents the comparison of spectra for the rhodamine solution (black curve) and PG impregnated with the same solution (red curve). The spectrum of the rhodamine solution has yellow fluorescence with a peak of around 550 nm and a half-width of ~40 nm. The rhodamine-impregnated PG possesses a red shift fluorescence with a peak around 575 nm as well as a long tail in the range from 580 to 650 nm with the corresponding half-width of ~35 nm. The red shift of the fluorescence spectrum indicates relatively different aggregation states of rhodamine molecules in PG compared to the solution. Moreover, the rhodamine-impregnated PG spectrum also has a non-symmetrical shape. We connect it with the overlap of the sample absorbance spectrum (dash line in Figure 5) and the registered fluorescence spectrum. A similar effect was observed in rhodamine-impregnated porous materials, such as sol–gel [32]. In addition, the spectrum curves are not smooth and have burrs, which originate from the measurement noise. Increasing the input laser power from 1 to 15 mW leads only to a spectral intensity change, keeping the shape of the curves unchanged.

**Figure 6:**
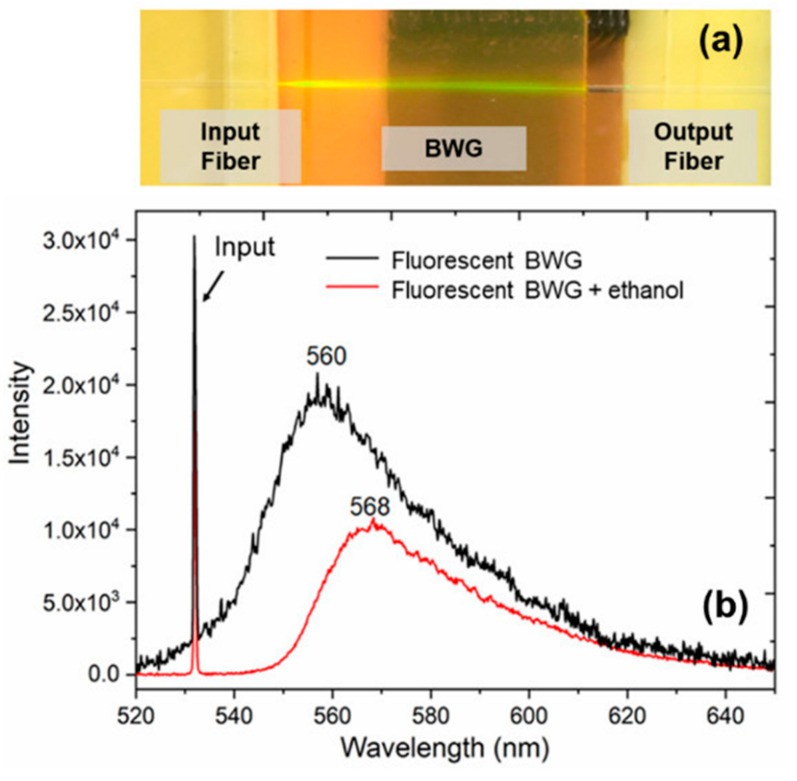
The image of laser radiation coupling into the BWG by the fiber–sample–fiber connection (**a**). The spectra curves of laser radiation transmitted through the BWG (**b**): black curve corresponds to the signal captured from the BWG in PG impregnated with rhodamine 6G and dried in the furnace (100 °C, 15 min), while the red curve demonstrates the same BWG after ethanol molecules (100%) captured by the nanoporous framework. Input radiation is 531.7 nm of the diode laser with power ~1.5 mW.

We would like to remind that before the BWG sensitivity testing, the sample is firstly dried in the furnace (100 °C, 15 min) to remove water and ethanol molecules from nanopores. Figure 6a shows an image of laser radiation coupling into the BWG by the fiber–sample–fiber connection. The spectrum of the BWG output light is given in Figure 6b (black curve), where the input green laser beam excites yellow fluorescence with a broadband of ~35 nm and a center at ~560 nm. It is reasonable to suppose that the rhodamine molecules immobilized around the core cause the fluorescence signal at the BWG output. A small dose of 4-μL ethanol liquid with a concentration of >99.9% was dropped on the same PG sample surface. The added liquid rapidly diffuses by the opened and interconnected nanopores. At least 6 s is required for the liquid to penetrate the BWG cladding. The response time was registered by the caption of near-field intensity distribution at the BWG output. We noticed that the added liquid mitigates the refractive index contrast between the core and cladding. However, that also decreases both the intensity of input radiation and yellow fluorescent light (Figure 6b, red curve). The noticeable shift of 8 nm was also observed after ethanol molecules penetrated the cladding. Such a red shift originates from the structural change of rhodamine molecules due to the introduction of a polar protic solvent—ethanol [32]. Besides, the obtained spectral curve becomes smoother and the amplitude of burrs is reduced. This results from the addition of ethanol liquid, which smooths the refractive index distribution of the BWG and suppresses the signal noise.

Next, we estimated the sensitivity of the BWG transducer by spotting the fluorescence peak wavelength shift while adding ethanol solutions with different concentrations. For the comparison, one more concentration of an ethanol–water solution with volume ratio of 1:1 was prepared and applied. The solution volume percentage of ethanol is ~ 50%. The refractive indices of the ethanol solution with concentrations of ~ 100% and ~ 50% are 1.3614 and 1.3598 at 20 °C at 589.29 nm [33], respectively.

**Figure 7:**
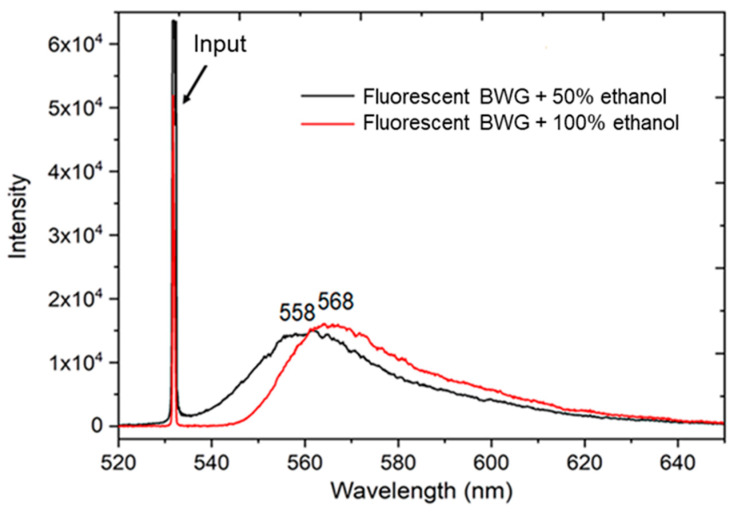
The spectra of laser radiation transmitted through the BWG: red curve corresponds to 100% ethanol deposited on the PG surface, while black curve corresponds to 50% ethanol solution. Input radiation is 531.7 nm with power ~3.0 mW.

Thus, 4-μL ethanol–water (50%) solution led to the peak shift of the fluorescence from ~ 568 to ~ 558 nm (Δλ ~ 10 nm) (Figure 7). The blue shift originates from the de-aggregation of rhodamine molecules due to the addition of water. The difference of the refractive indices between these two solutions is Δn = 0.0016. Therefore, the sensitivity of the peak shift of the fluorescence spectrum to the refractive index of the solution is preliminarily quantified as 6250 ± 150 nm/RIU (where RIU is a refractive index unit), which is calculated by the ratio S = Δλ/Δn_s_ [34]. This value is ~10 times higher compared with the sensitivity obtained with the porous silicon waveguide sensor (560 ± 50 nm/RIU) [34].

**Figure 8:**
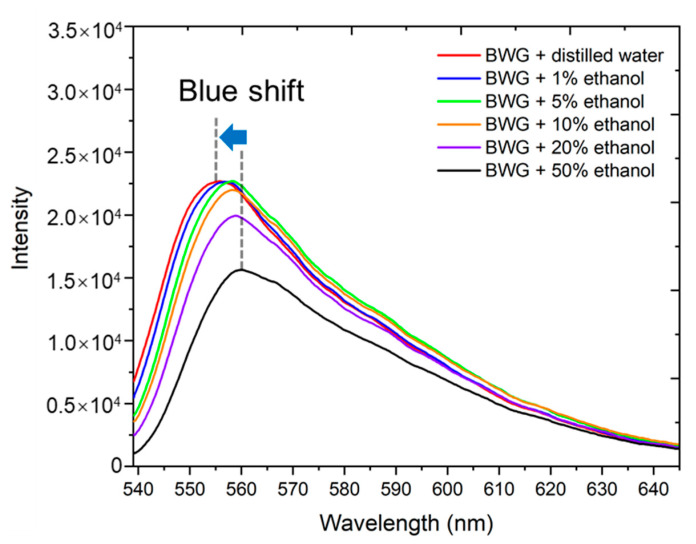
The spectra of laser radiation transmitted through the BWG with ethanol solutions deposited on the PG surface. The concentrations by volume of the ethanol solutions are 50%, 20%, 10%, 5%, and 1%, and 0% indicates distilled water.

To find the detection threshold of the BWG transducer, we registered the output spectra after the deposition of the ethanol–water solution with various concentrations (by volume): 50%, 20%, 10%, 5%, and 1% (Figure 8). The results are also compared with the deposition of distilled water. As a result, since the ethanol concentration decreases, the fluorescence spectrum blue shifts and the intensity of fluorescence increases. Decreasing the concentration less than 1% did not allow for distinguishing the difference in the output spectrum—it coincided with the distilled water spectrum. Therefore, the detection threshold is 1%.

### 3.3. Single-Line Emission from the BWG

**Figure 9:**
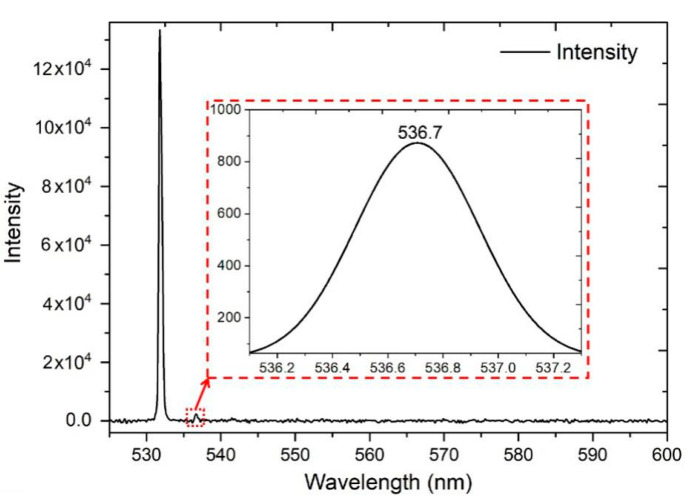
The spectrum of laser radiation transmitted through the BWG impregnated with rhodamine and containing ethanol molecules in the cladding. The input power is 10 mW. The inserted image is the enlarged spectrum of the peak at 536.7 nm with an FWHM (full width at half maximum) bandwidth of ~0.5 nm.

During the experiment, we noticed that the increase in the input laser power up to 10 mW results in fluorescent peak narrowing and appearance of a single-line emission peak with position at 536.7 nm and an FWHM bandwidth of ~0.5 nm (Figure 9). This peak occurs only when ethanol molecules penetrate the rhodamine containing the BWG cladding. The peak was observed neither in the initial rhodamine solution nor in the BWG in PG impregnated with rhodamine at a similar or even much higher input laser power.

**Figure 10:**
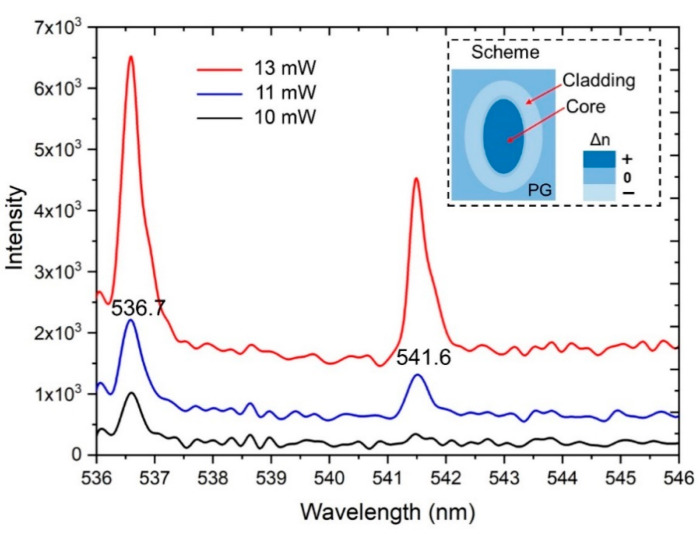
The spectra of laser radiation transmitted through the BWG impregnated with rhodamine and containing ethanol molecules (100%) in the cladding at input powers of 10 (red curve), 11 (blue curve), and 13 mW (black curve). The peaks at 536.7 and 541.6 nm possess an FWHM bandwidth of ~0.5 nm. The inserted image is a schematic diagram of the BWG refractive index distribution in the cross-section.

The following increase in the input power up to 11 and 13 mW allowed us to detect another peak at 541.6 nm with the same FWHM bandwidth of ~0.5 nm (Figure 10). The difference between the two peaks is 4.9 nm. The intensities of these peaks increase with higher input power. A similar phenomenon was recently reported using a silica fiber with a diameter of 125 μm in a rhodamine–ethanol solution [35], where the appearance of the second emission peak was connected with a transformation from a single-mode to multi-mode emission when increasing the input radiation power over the threshold value. Our BWG is schematically shown in Figure 9 and consists of the densified core and rarefaction cladding. The fluorescent molecules are regarded to be immobilized in the cladding and excited by the evanescent wave of the guiding light. Based on the analysis from [35], the spacing between two peaks can be estimated by λ^2^/πnD = 5.7 nm, where n ~ 1.34 for the BWG in PG [24] and D is the diameter of the BWG (equal to the BWG height of ~12 μm); this is close to the value obtained from the experimental result of 4.9 nm. It is also important to note that only narrow peaks with an FWHM up to 0.5 nm were observed in our experiments.

## 4. Conclusions

In this study, we have designed, fabricated, and tested the novel configuration of a PG-based sensor. Specifically, we inscribed an optical micro-sized channel—a BWG—in PG, which functions as the primary transducer of the sensor interrogating the internal conditions of the nanoporous material, and detects the chemical reactions occurring inside nanopores. The transducer showed the principal ability to detect target small molecules, such as ethanol, which were deposited on the PG surface. The detection threshold of volume concentration is equal to 1%. The sensitivity of the peak shift of the fluorescence spectrum to the refractive index of the solution was quantified as 6250 ± 150 nm/RIU.

It is important to highlight features which we revealed during the BWG transducer operation. The BWG cladding has enough space for the indicator (rhodamine 6G) and target molecules (ethanol). The coupled laser radiation excited the rhodamine 6G molecules, which generated yellow fluorescence registered at the waveguide output. The reaction of rhodamine 6G and ethanol molecules caused the shift of the fluorescence spectrum (~10 nm). In the future, such ability to detect small molecules can be utilized for the sensor industry and lab on a chip applications.

Therefore, at relatively high input power, additional single-line peaks at the BWG output were registered. Such peaks occur only when ethanol molecules penetrate the rhodamine-containing BWG cladding. The following increase in the input power up to 11 and 13 mW allowed us to detect one more peak at ~541 nm with the same FWHM bandwidth of ~0.5 nm.

We have also obtained the results for the LDW of BWGs in PG, which are promising for the laser scientific community. The previously proposed mechanism of PG densification by femtosecond laser pulses [23] was optimized for the formation of the core–cladding BWG type of an elongated ellipse-shape (12 × 5 µm) and with the advantage of moderate optical losses. In addition, the array of BWGs with 40 channels (10 μm spacing) was fabricated with low cross-talk <5%. This result is important from the point of view of prospects for spatial integration in PG, opening the opportunity to fabricate arrayed BWGs with low light cross-talk between neighbor waveguides in a single glass plate.
